# *Schistosoma haematobium* effects on *Plasmodium falciparum* infection modified by soil-transmitted helminths in school-age children living in rural areas of Gabon

**DOI:** 10.1371/journal.pntd.0006663

**Published:** 2018-08-06

**Authors:** Jean Claude Dejon-Agobé, Jeannot Fréjus Zinsou, Yabo Josiane Honkpehedji, Ulysse Ateba-Ngoa, Jean-Ronald Edoa, Bayodé Roméo Adegbite, Ghyslain Mombo-Ngoma, Selidji Todagbe Agnandji, Michael Ramharter, Peter Gottfried Kremsner, Bertrand Lell, Martin Peter Grobusch, Ayôla Akim Adegnika

**Affiliations:** 1 Centre de Recherche Médicales de Lambaréné (CERMEL) and African Partner Institution, German Center for Infection Research, Lambaréné, Gabon; 2 Center of Tropical Medicine and Travel Medicine, Department of Infectious Diseases, Division of Internal Medicine, Academic Medical Center, University of Amsterdam, Amsterdam, the Netherlands; 3 Department of Parasitology, Leiden University Medical Center, Leiden, the Netherlands; 4 Departement de Parasitologie-Mycologie, Faculté de Médecine, Université des Sciences de la Santé, Owendo, Gabon; 5 Institute of Tropical Medicine, University of Tübingen and Partner site Tübingen, German Center for Infection Research, Tübingen, Germany; 6 Department of Tropical Medicine, Bernhard Nocht Institute for Tropical Medicine & I, Department of Medicine, University Medical Centre, Hamburg-Eppendorf, Hamburg, Germany; George Washington University, UNITED STATES

## Abstract

**Background:**

Malaria burden remains high in the sub-Saharan region where helminths are prevalent and where children are often infected with both types of parasites. Although the effect of helminths on malaria infection is evident, the impact of these co-infections is not clearly elucidated yet and the scarce findings are conflicting. In this study, we investigated the effect of schistosomiasis, considering soil-transmitted helminths (STH), on prevalence and incidence of *Plasmodium falciparum* infection.

**Methodology:**

This longitudinal survey was conducted in school-age children living in two rural communities in the vicinity of Lambaréné, Gabon. Thick blood smear light microscopy, urine filtration and the Kato-Katz technique were performed to detect malaria parasites, *S*. *haematobium* eggs and, STH eggs, respectively. *P*. *falciparum* carriage was assessed at inclusion, and incidence of malaria and time to the first malaria event were recorded in correlation with Schistosoma carriage status. Stratified multivariate analysis using generalized linear model was used to assess the risk of plasmodium infection considering interaction with STH, and survival analysis to assess time to malaria.

**Main findings:**

The overall prevalence on subject enrolment was 30%, 23% and 9% for *S*. *haematobium*, *P*. *falciparum* infections and co-infection with both parasites, respectively. Our results showed that schistosomiasis in children tends to increase the risk of plasmodium infection but a combined effect with *Trichuris trichiura* or hookworm infection clearly increase the risk (aOR = 3.9 [_95%_CI: 1.7–9.2]). The incidence of malaria over time was 0.51[_95%_CI: 0.45–0.57] per person-year and was higher in the Schistosoma-infected group compared to the non-infected group (0.61 *vs* 0.43, *p* = 0.02), with a significant delay of time-to first-malaria event only in children aged from 6 to 10-years-old infected with *Schistosoma haematobium*.

**Conclusions:**

Our results suggest that STH enhance the risk for *P*. *falciparum* infection in schistosomiasis-positive children, and when infected, that schistosomiasis enhances susceptibility to developing malaria in young children but not in older children.

## Author summary

Despite the progress made in the last decade, malaria remains a serious public health issue in sub-Saharan region, where it overlaps with helminths infections. The interactions between both are manifold and complex, requiring further investigation. We report here that *Trichuris trichiura* or hookworm associated with schistosomiasis increase the risk of *Plasmodium falciparum* infection; whilst schistosomiasis is independently associated with a malaria increase in young children, but not in the older children. Our finding is an additional evidence that optimizing helminth control mainly in children contributes to overcoming malaria in areas endemic for both parasitic infections where children are those who bear the highest burden of these infections.

## Introduction

Over the past fifteen years, morbidity and mortality due to malaria have globally decreased. However, sub-Saharan Africa, where 90% cases of malaria and 92% of deaths related to malaria have occurred in 2015, still bears the highest burden of the disease [1]. Most of these cases remain confined to rural and semi-urban areas [2–4] where helminths are co-prevalent [5–7]. In these areas where malaria significantly overlaps with helminth infections, several studies have reported interactions between the two parasitic infections at both immunological [8–12] and epidemiological [13–16] levels. Studies have reported an effect of helminths on the cellular and humoral immune responses to the malaria parasites mainly in children [8–12,17]. Some authors have reported that this effect leads to the aggravation of clinical manifestations of malaria. Indeed, it has been shown that *Trichuris trichiura* infection was associated with increased malaria prevalence, while an increased helminth burden was associated with increased *Plasmodium falciparum* or *Plasmodium vivax* parasitemia [18], or enhanced anemia in co-infected children [19]. Another author has reported a positive effect of helminths on malaria outcomes. Indeed, Nacher et al found that helminths, particularly Ascaris, may have a role in the establishment of malaria tolerance in Thai patients [20]. However HIV co-infection complicated the picture further. Indeed, high prevalences of helminth infections and malaria have been reported in HIV positive people, particularly in pregnant women under anti-retroviral therapy (ART) in Rwanda [21–23] with 10% co-infections with both [23]. These high infection prevalences are found to be associated with a low CD4 counts and moreover, each of these infections is a risk factor for the other [22].

The situation is similarly unclear when it comes to *Schistosoma* spp. infections. It has been reported that the effect of schistosomiasis on malaria may depend on the *Schistosoma* species [24], or may be conflicting even for the same species [15,25–27]. Indeed, some reports have indicated that infection with *S*. *haematobium* can confer protection against severe malaria in children [25], reducing the risk of progression to symptomatic disease in long-term asymptomatic carriers of *P*. *falciparum* [15], or can delay the occurrence of a malaria episode in children [26]; whereas others found that *S*. *haematobium* may increase the prevalence of *P*. *falciparum* parasites in co-infected children [27]. In contrast, *Schistosoma mansoni* was reported to significantly increase the malaria incidence rate in children [28]. These finding provide evidence of the effect of schistosomiasis on *Plasmodium* infection. Current results on schistosomiasis and plasmodial co-infection are conflicting as reviewed recently by Adegnika et al. [24]. Most studies conducted to address these co-infections are cross-sectional in nature and could be limited in their capacity to precisely examine interactions between schistosomiasis and malaria. In this study, we conducted a longitudinal survey in order to address this issue in an area where *S*. *haematobium* and *P*. *falciparum* are the main prevalent species of schistosomiasis and malaria [13,14]. We thus assessed the effect of *S*. *haematobium* on clinical and parasitological aspects of *P*. *falciparum* infection in school aged children living in this co-endemic area, including the effect of soil-transmitted helminths (STH) in this association.

## Methods

### Ethics statement

The study was approved by the institutional ethics committee of CERMEL, reference number: CEI-MRU 002/2012. Parents or legal representatives of the participant gave a written informed consent. The study was conducted in line with the Good Clinical Practice (GCP) principles of the International Conference on Harmonisation (ICH) [29] and the Declaration of Helsinki [30].

### Study site

The study took place at CERMEL (Centre de Recherches Médicales de Lambaréné). Data and samples were collected from May 2012 to December 2014 in Bindo-Makouké villages (BM) and Zilé-PK villages, two settlements in the vicinity of Lambaréné [14] situated approximately at 60 km and 120 km, respectively, to the South of the Equator. The rainfall is perennial except for the long dry season (from June to September) with a mean of 1,216 mm per year [31]. The region is irrigated by the Ogooué River and its tributaries, with many ponds, lakes and streams constituting favourable conditions for fresh-water snail habitation. Recent published data demonstrate that the prevalence in the area for *S*. *haematobium* range from 15% to 75% [9,13,14]. Water supply, fishing, household work, fetching water and playing are some activities which expose the local population to schistosomiasis. Malaria transmission is perennial and the dominant malaria parasite species is *P*. *falciparum* [32,33].

### Study design

The study was designed as a prospective longitudinal study.

### Study population and inclusion criteria

School-age children living in two vicinities of Lambaréné (Nzilé-PK villages and Bindo-Makouké villages) were invited to participate in the study. Volunteers without any known chronic diseases other than possible helminth co-infections, and living in the study area for at least one year before inclusion were eligible to take part to the study. During the survey, participants found with a recurrent or severe disease other than malaria or helminth infection were excluded from the study.

### Sample size determination

A previous study conducted in the vicinities of Lambaréné reported a 42% prevalence for plasmodium infection among school age children [9]. To be able to detect a minimum of 12.5% prevalence difference of plasmodium infection between children with schistosomiasis and those without, with a minimum of 80% power, we needed to include in the study at baseline at least 249 children for each study group, giving a total of 498 volunteers school age children.

### Study procedure

Field-workers went to each house and school of both villages to invite through their parents or legal representatives school-age children to participate in the study. Eligible and consenting volunteers were included. At baseline, demographics (age, sex and location) and anthropological (weight, height) data were collected. Axillary temperature was recorded. *S*. *haematobium* infection, *P*. *falciparum* infection and soil-transmitted helminths (STH) status were assessed. Participants were treated if they were found to harbour either of those parasitic infections. The follow-up consisted of two kinds of visit: active visits consisted of monthly home visits for any malaria-like symptoms assessment and recording of any medication intake; and passive visits were ad-hoc presentations of participating children at the research centre for any health issues, including flu-like symptoms.

Malaria status was defined as positive thick blood smear (TBS) associated with fever, or history of fever in the past 48h from the time of visit. Fever was defined as an axillary temperature of 37.5°C or higher. In case malaria was diagnosed, urine filtration was performed to assess evidence of co-infection with urogenital schistosomiasis. Urine filtration was also performed during the follow-up every time the children had visible haematuria.

Study groups were determined based on the schistosomiasis status. This was done differently for baseline analysis and for longitudinal analysis. At baseline and for baseline analysis, participants found infected with *S*. *haematobium* were assigned to the ‘Schistosoma-positive’ (S^+^) group and the others to the ‘Schistosoma-negative’ (S^-^) group. For longitudinal analysis, study groups were formed at the end of the follow-up period, and we assigned any participants found with infection at baseline and at any time point of the study course to the ‘Schistosoma-infected’ (SI) group. Those found negative at baseline and who did not experienced schistosomiasis during the study course were assigned to the ‘Schistosoma-uninfected’ (SU) group. Time of exposure to malaria infection for incidence calculation did not include the first 28 days after each malaria treatment.

In accordance with the national guidelines, treatment of schistosomiasis consisted of the administration of 40 mg of praziquantel per kilogram body weight once; asymptomatic *P*. *falciparum* parasitemia and malaria episodes were treated with tablets of 20/120mg of artemether-lumefantrine combination therapy given according to the body weight twice a day, in three consecutive days. Treatment of STH was a once-daily dose of 400 mg of albendazole for three consecutive days [34]. For any other cause of a disease episode, the participant was referred to the appropriate health centre.

### Samples collection and laboratory assays

Detection of malaria parasites was done microscopically by TBS using the Lambaréné method as described elsewhere [35–37]. Detection of *S*. *haematobium* eggs was done by filtration of 10ml of fresh urine using a 12μm pore-size filter as previously described [38–40]. For the diagnosis of urogenital schistosomiasis, urine samples were collected over three consecutive days, unless the participant was found positive with at least one parasite egg in any sample before the second or the third day. The Kato-Katz technique was performed to assess the presence of *A*. *lumbricoides*, *T*. *trichiura* and hookworm in fresh stool samples [41]. For each time point of STH assessment, one stool sample was collected. For each stool sample, two slides were performed and each slide was independently read by two readers.

### Statistical analysis

Data were captured on the patient report form (PRF), entered in Access 2013 software and transferred to R software version 3.2.4 for analysis. Univariate and multivariate analysis were performed applying the Generalized Linear Model (GLM). For multivariate analysis, first we considered the interaction between asymptomatic *P*. *falciparum* infection as the main variable and each explanatory variable. In case of effect measure modification, the analysis was stratified on the variable, and the Breslow test was done to assess the homogeneity of the strata. Otherwise, the variable was evaluated as confounding factor to be include in the final model. Ten per cent (10%) or more difference of estimated measure of association before and after adjustment was used to define confounding factors. The effect of STH infection was assessed separately with respect to the species. Incidence of malaria was estimated in person-year according to each variable. A Kaplan Meier curve was drawn to assess time-to-malaria occurrence. The Log-rank test was used to compare the curves and the Cox model was used for adjusted analysis.

## Results

### Study population at baseline

**Fig 1:**
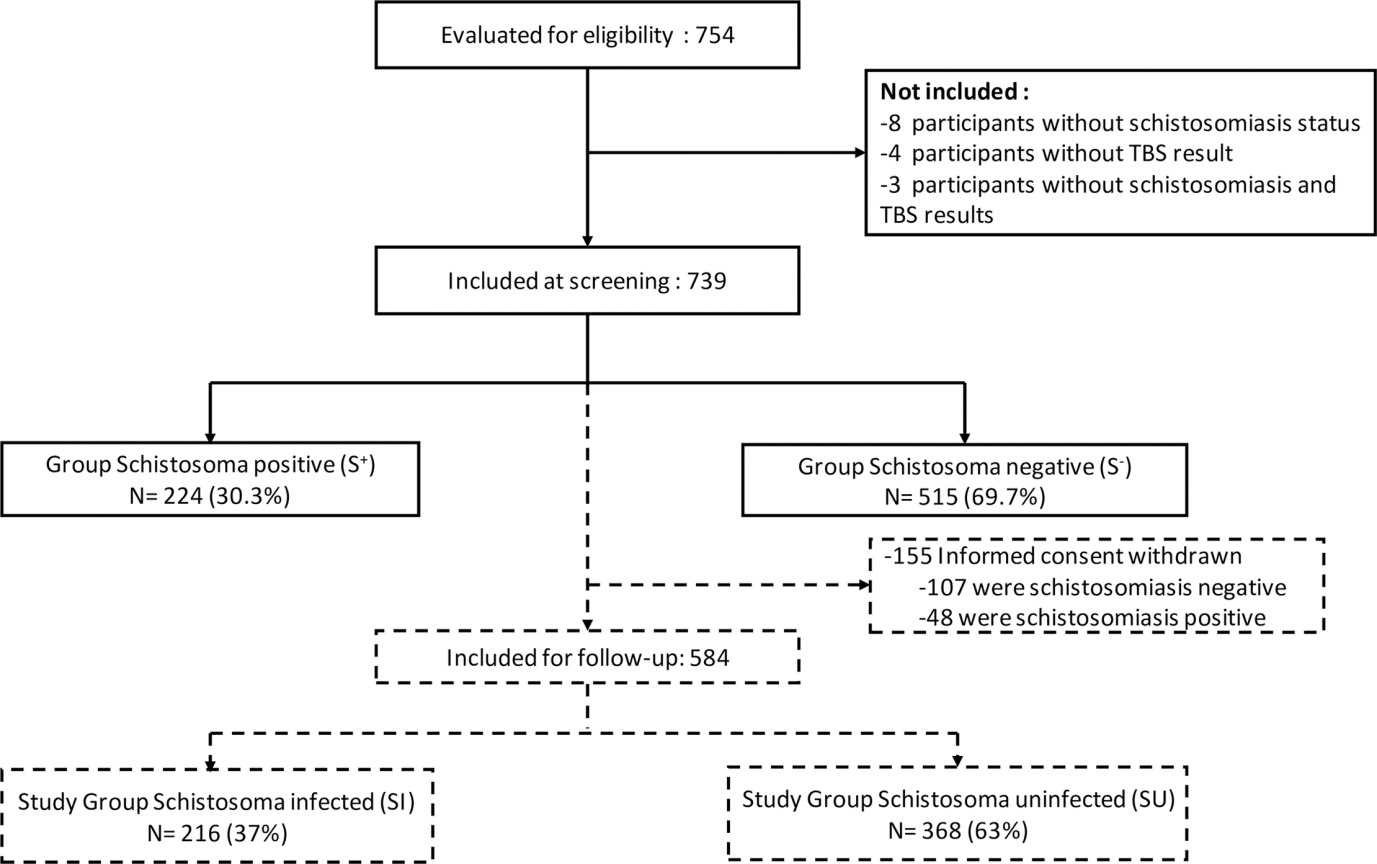
Flow chart of the participants during the study course. The inclusion phase is shown as a solid line and the follow-up phase is shown as a broken line.

Among the participants who were invited to participate in the study, informed consent was granted for 754 children by their parents or their legal representatives. Of those, a total of 739 children with schistosomiasis and *P*. *falciparum* status available were included at baseline (Fig 1). Among participants enrolled, 68 (9%) children were not able to provide sample stool at baseline and from the others, 31% [_95%_CI: 27%-35%] were infected with STH. The most prevalent infection was trichuris with 21% [_95%_CI: 18%-24%] followed by ascaris and hookworm with 19% [_95%_CI: 16%-22%] and 6% [_95%_CI: 5%-8%], respectively. Mean age of these study population was 10.4 (SD = 3.1) years, the boy-to-girl sex ratio was 1.1:1 (Table 1). Of participants included, 586 (79%) agreed to be followed-up for malaria incidence.

**Table 1:** Characteristic of 739 participants seen at inclusion.

		Study population		
		n	(%)	_95%_CI(%)
**Age**				
	**[6–10]**	382	(51.7)	[48.0–55.3]
	**[10–16]**	357	(48.3)	[44.6–52.0]
**Age (mean, sd)**		(10.4, 3.1)	/	/
**Sex**				
	Female	351	(47.5)	[43.8–51.2]
	Male	388	(52.5)	[48.8–56.1]
**Sex ratio (M/F)**		(1.1)		
**Location**				
	Bindo-Makouké villages	420	(56.8)	[53.2–60.4]
	Zilé-PK villages	319	(43.2)	[39.6–46.8]
***S*. *haematobium* infection**				
	Negative	515	(69.7)	[66.2–73.0]
	Positive	224	(30.3)	[27.0–33.8]
***P*. *falciparum* infection**				
	Negative	572	(77.4)	[74.2–80.4]
	Positive	167	(22.6)	[19.6–25.8]
Soil-transmitted helminths*				
	*A*. *lumbricoides*	127	(18.9)	[16.0–22.1]
	*T*. *trichiura*	139	(20.7)	[17.7–24.0]
	Hookworm	43	(06.4)	[04.7–08.5]
	Any STH	208	(31.0)	[27.5–34.6]

*68 missing data

### Distribution of *P*. *falciparum* and *S*. *haematobium* infections

The prevalence of *P*. *falciparum* and *S*. *haematobium* at baseline was 23% [_95%_CI: 20%-26%] and 30% [_95%_CI: 27%-34%], respectively; with 67 (9%, [_95%_CI: 07%-11%]) participants co-infected with both parasites. Eight per cent of participants infected with *P*. *falciparum* had fever. As shown in Table 2, both infections were more prevalent in Zilé-PK villages compared to Bindo and Makouké villages with 29% [_95%_CI: 24%-34%] *vs* 18% [_95%_CI: 14%-22%], respectively, for *P*. *falciparum* and 45% [_95%_CI: 40%-51%] *vs* 19% [_95%_CI: 15%-23%], respectively, for *S*. *haematobium*. The prevalence of both infections was similar for age and sex groups.

**Table 2:** Distribution of *P*. *falciparum* and *S*. *haematobium* infections among the 739 participants seen at baseline.

		*P*. *falciparum* infection			*S*. *haematobium* infection		
		n	% [_95%_CI(%)]	*p*	n	% [_95%_CI(%)]	*p*
**Total**		167	22.6 [19.6–25.6]	/	224	30.3 [27.1–33.7]	/
**Sex**				0.16			0.74
	Female	71	20.2 [16.3–24.8]		104	29.6 [25.1–34.6]	
	Male	96	24.7 [20.7–29.3]		120	30.9 [26.5–35.7]	
**Age group**				0.93			0.17
	[6–10]	87	22.8 [18.8–27.0]		107	28.0 [23.7–32.7]	
	[10–16]	80	22.7 [18.4–27.0]		117	32.8 [28.1–37.8]	
	(Mean,sd)	(10.1, 3.1)			(10.7, 3.0)		
**Location**				<0.001			<0.001
	Bindo-Makouké villages	75	17.9 [14.5–21.8]		80	19.0 [15.6–23.1]	
	Zilé-PK villages	92	28.8 [24.1–34.0]		144	45.1 [39.8–50.6]	
STH*							
	*Ascaris*	30	23.6 [16.5–32.0]	0.88	43	33.9 [25.7–42.8]	0.52
	*Trichuris*	41	29.5 [22.1–37.8]	0.05	49	35.2 [27.3–43.8]	0.28
	Hookworm	11	25.6 [13.5–41.2]	0.70	21	48.8 [33.3–64.5]	0.13
	Any STH	52	25.0 [19.3–31.5]	0.43	72	34.6 [28.2–41.5]	0.24

*68 missing data

### Study group characteristics

Among the participants followed-up for malaria incidence assessment, 216 (37%) were found positive for Schistosoma infection during the study period, including 176 cases on inclusion and additionally 40 new cases during follow-up and assigned to SI group. The 368 (63%) others participants who remained negative during the whole study course were thus assigned to the SU group (Fig 1). As shown in Table 3, the two study groups were comparable for all parameters except for location and *P*. *falciparum* parasite carrier status. Indeed, 168 (78%) of SI children came from Zilé-PK villages while 253(69%) of SU children came from Bindo and Makouké villages *(p*<0.001). Additionally, prevalence of *P*. *falciparum* parasite carriage was significantly higher in the SI group compared to the SU group (31% *vs* 20%, *p* = 0.004).

**Table 3:** Characteristics of study groups considered for longitudinal analysis regarding Schistosoma status (N = 586).

		Schistosoma Infected (n = 216)		Schistosoma Uninfected (n = 368)		*P*
		n	% [_95%_CI (%)]	n	% [_95%_CI (%)]	
**Sex**						0.49
	Female	111	51.4 [44.5–58.2]	177	48.1 [42.9–53.3]	
	Male	105	48.6 [41.8–55.5]	191	51.9 [46.7–57.1]	
**Age**						0.14
	[6–10]	110	50.9 [44.0–57.8]	211	57.3 [52.1–62.4]	
	[10–16]	106	49.1 [42.2–55.9]	157	42.7 [37.5–47.9]	
	n, (mean, sd)	216	(10.4, 3.1)	368	(10.1, 3.0)	0.19
**Location**						< 0.001
	Bindo-Makouké villages	48	22.2 [16.9–28.4]	253	68.7 [63.7–73.4]	
	Zilé-PK villages	168	77.8 [71.6–83.1]	115	31.3 [26.5–36.3]	
***P*. *falciparum* parasite carriage**						0.004
	Positive	66	30.6 [24.5–37.2]	74	20.1 [16.1–24.6]	
**STH species**						
	*A*. *lumbricoïdes*	30	13.9 [09.6–19.2]	60	16.3 [12.7–20.5]	0.47
	*T*. *trichiura*	38	17.6 [12.8–23.3]	58	15.8 [12.2–19.9]	0.56
	Hookworm	17	07.9 [04.6–12.3]	14	03.8 [02.1–06.3]	0.05
	Any species	58	26.9 [21.1–33.3]	97	26.4 [21.9–31.2]	0.92

### Association between *S*. *haematobium* and *P*. *falciparum* parasitic infections

At crude analysis as given in Table 4, *Schistosoma* infection (*p* = 0.002) and location (*p*<0.001) were associated with *P*. *falciparum* infection. Children infected with *S*. *haematobium* had a 1.8 [_95%_CI: 1.2–2.5] times odds of being co-infected with *P*. *falciparum* parasites compared to their non-infected counterpart. After adjustment (Table 4), *P*. *falciparum* infection remains associated only with location (*p* = 0.02) while a trend of association with schistosomiasis infection was found (*p* = 0.06).

**Table 4:** Potential risk factors including *S*. *haematobium* infection associated with *P*. *falciparum* infection among the 739 participants seen at baseline.

		Crude analysis		Adjusted analysis	
		OR [_95%_CI(OR)]	*p*	aOR [_95%_CI(aOR)]	*p*
***S*. *haematobium* status**			0.002		0.06
	Negative	1		1	
	Positive	1.8 [1.23–2.53]		1.5 [0.98–2.18]	
**Location**			<0.001		0.02
	Bindo-Makouké villages	1		1	
	Zilé-PK villages	1.9 [1.32–2.64]		1.6 [1.09–2.37]	
**Sex**			0.14		0.38
	Female	1		1	
	Male	1.3 [0.92–1.84]		1.2 [0.82–1.72]	
**Age (years)**		1.1 [0.99–1.11]	0.09	0.9 [0.89–1.01]	0.08
***T*. *trichiura***			0.06		0.09
	Negative	1		1	
	Positive	1.5 [0.98–2.28]		1.5 [0.94–2.38]	
***A*. *lumbricoïdes***			0.93		0.72
	Negative	1		1	
	Positive	1.0 [0.64–1.59]		0.9 [0.55–1.49]	
**Hookworm**			0.72		0.76
	Negative	1		1	
	Positive	1.1 [0.54–2.25]		0.9 [0.39–1.88]	

In the following analysis, we found effect modification of *Trichuris* or hookworm infections on *P*. *falciparum* and *S*. *haematobium* infections association. As presented in Table 5, analysis stratified on those two infections showed that among study participants without *T*. *trichiura* and hookworm infections, there is no effect of *S*. *haematobium* on *P*. *falciparum* parasite carriage; while among those infected with either hookworm or *T*. *trichiura* or a combination, children co-infected with *S*. *haematobium* had a 3.1 ([_95%_CI: 1.5–6.4], *p* = 0.002) time odds of being infected with *P*. *falciparum*. This finding remained statistical significant when adjusted for age, sex, ascariasis and location (aOR = 3.9 [_95%_CI: 1.75–9.19], *p* < 0.001). Age, sex and *Ascaris* infection were forced in the final model of the GLM analysis.

**Table 5:** Association between asymptomatic *Plasmodium falciparum* infection and *Schistosoma haematobium* infection stratified on *Trichuris trichiura* and hookworm infections among the 671 participants with known STH infection status and seen at baseline.

			N	Crude analysis^+^		Adjusted analysis*	
				OR [_95%_CI(OR)]	*p*	aOR [_95%_CI(aOR)]	*p*
***T*. *trichiura* and hookworm negative (N = 516)**							
	***S*. *haematobium* status**				0.27		0.84
		Negative	360	1		1	
		Positive	156	1.3 [0.83–2.01]		1.1 [0.65–1.67]	
***T*. *trichiura* or hookworm positive (N = 155)**							
	***S*. *haematobium* status**				0.002		<0.001
		Negative	100	1		1	
		Positive	55	3.1. [1.48–6.44]		3.9 [1.75–9.19]	

^+^Breslow-test, *p* = 0.046

*Adjusted for location, sex, age and *A*. *lumbricoïdes* infection

### Effect of *S*. *haematobium* Infection on *P*. *falciparum* malaria incidence

During the 19 months follow-up phase for *P*. *falciparum* malaria incidence assessment, 210 (36%) participants had developed a total of 318 new cases of malaria (Table 6). The overall incidence was 0.51 [_95%_CI: 0.47–0.55] per person-year. Taking into account the study groups, participants in the SI group had 1.4 [_95%_CI: 1.1–1.8] times the risk of developing malaria compared to their counterparts in the SU group.

**Table 6:** Malaria risk and malaria incidence among the 584 participants according to Schistosoma status and other risk factors.

Study group		Number of participants exposed	Participants who developed malaria attack		Malaria attack cases				
			n (%)	RR [_95%_CI(RR)]	Number of cases	Exposure Time ^+^	Incidence [_95%_CI(I)]	RR [_95%_CI(RR)]	*P-value*
**Total**		584	210 (36.0)	/	318	627	0.51 [0.47–0.55]	/	
**Schistosoma status**									0.002
	Uninfected	365	109 (29.9)	1	162	373	0.43 [0.38–0.48]	1	
	Infected	219	101 (46.1)	1.54 [1.17–2.02]	156	254	0.61 [0.55–0.67]	1.42 [1.14–1.77]	
**Location**									0.76
	Bindo-Makouké villages	301	107 (35.5)	1	163	316	0.51 [0,45–0,56]	1	
	Zilé-PK villages	283	103 (36.4)	1.02 [0.78–1.34]	155	311	0.50 [0,44–0,55]	0.98 [0.79–1.22]	
**Age**									0.73
	[6–10]	321	123 (38.3)	1	178	345	0.52 [0.47–0.57]	1	
	[10–16]	263	87 (33.1)	0.86 [0.65–1.13]	140	282	0.50 [0.44–0.56]	0.96 [0.77–1.20]	
**Sex**									0.21
	Female	288	97 (33.7)	1	143	304	0.47 [0.41–0.53]	1	
	Male	296	113 (38.2)	1.13 [0.86–1.48]	175	323	0.54 [0.48–0.60]	1.15 [0.92–1.43]	

^+^In person-year

**Fig 2:**
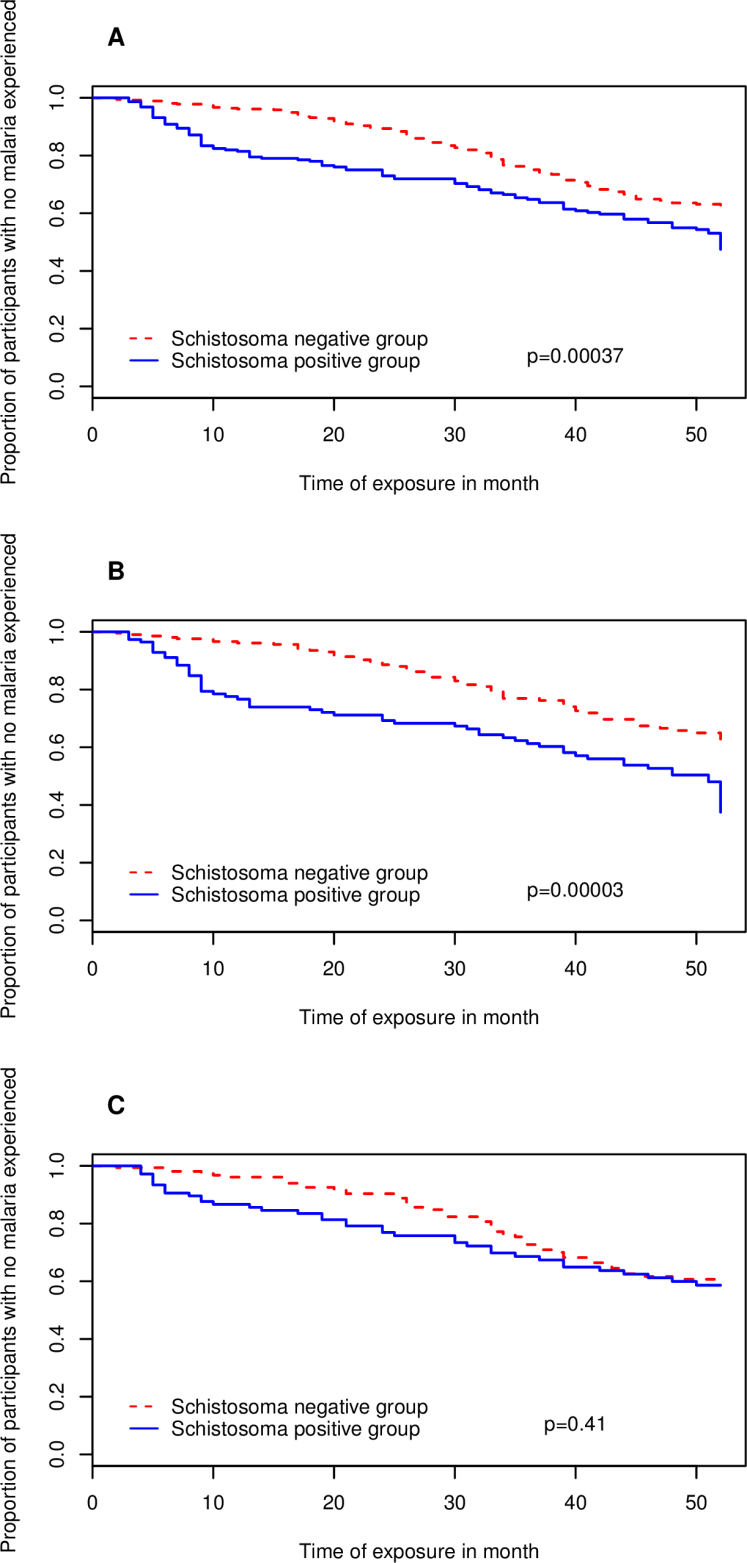
Depicts estimates of time to malaria after 52 weeks of follow-up. Depicted in the vertical axis the proportion of children who did not experience malaria and in the horizontal axis, the follow-up time in months. In red, children in *S*. *haematobium* non-infected group and in blue children in *S*. *haematobium* infected group. 2A) Kaplan Meier curve for time-to-first malaria case for overall study population. 2B) Kaplan Meier curve for time-to-first malaria case for children aged from 6 to 10 years old. 2C) Kaplan Meier curve for time-to-first malaria case for children aged from 11 to 16 years old.

The time-to-first malaria episode was assessed for the first twelve months of follow-up of each participant and those who did not develop malaria before the end of that time were censored. Our results show that in the SI group, among the 101 (46%) participants developed malaria, the median time to first malaria episode was 52 weeks. For their counterparts of SU group where 109 (30%) participants developed malaria, the median time to first malaria episode was not reached at the end of 52 weeks of follow-up. As presented in Fig 2, we found a significant delay to malaria in the SU group compared to SI group (Log-Rank test: *p* = 0.00037). Assessing the delay until development of malaria according to schistosomiasis status, we found that SI group participants had a 1.6 (Cox model: *p* = 0.0004) times increased risk of early development of malaria as compared to their counterparts of the SU group. This association remains significant when adjusted for location, age and sex (aRR = 1.9, *p* = 0.000034). Stratifying for age yielded a significant delay in time-to-first-malaria episode in the SU group (median time not reach) compared to the SI group, where the median time was 51 weeks (*p* = 0.00003) for 6–10 year old children. Children with schistosomiasis had a 2.1 (Mantel-Cox test: *p* = 0.00004) times increased risk of developing malaria compared to children without schistosomiasis. On the contrary, there was no difference in terms of delay in time-to-first malaria episode between the two study groups (*p* = 0.41) in children aged 11–16 years.

## Discussion

In area where schistosomiasis is endemic, the question of its effect on malaria outcome is a growing concern. Our study area is endemic for both infections [14], and our study reveals that up to 11% of school-age children could be co-infected with *P*. *falciparum* and *S*. *haematobium* parasites, comparing to up to 15% of pregnant women co-infected in Cameroon [42] or 23% of children co-infected with *S*. *mansoni* in a co-endemic area of North-Western Tanzania [43]. In our study area, poly-parasitism is evident [8,9,13,14,24]. The prevalence of STH species ranged from 32% to 48% among children infected with *S*. *haematobium*. This finding is not surprising since STH is commonly reported to be prevalent in rural areas [43]. Therefore, the risk to be infected by multiple parasites including *S*. *haematobium* is high [44]. Since these intestinal parasites are known to be able to modulate the immune system of the host, it would be necessary to assess the effect of these infections on Schistosoma-*P*. *falciparum* association.

Everything else equal, we found a trend of association between risk of *P*. *falciparum* carriage and Schistosoma infection. In univariate analysis, Adedoja et al. found that children infected with *S*. *heamatobium* have equal chances of being infected with *P*. *falciparum* as children with no worm infection [45], while Ateba and collaborators found a significant increase of plasmodium asexual parasite prevalence among Schistosoma infected children in comparison to the uninfected [9]. Conflicting results found in the literature on schistosomiasis-malaria co-infection issue suggest that there are potential confounding factors not yet established which need to be taken into account. In this study, we found an effect-measure modification of *Trichuris* and Hookworm infections on the association between *S*. *haematobium* and *P*. *falciparum*. As well, location was identified as confounding factor. Some authors have previously shown that *T*. *trichiura* [18] and hookworm [45,46] individually can affect the association between schistosomiasis and malaria co-infection by increasing the risk of being infected with *P*. *falciparum* parasites. Our analysis was stratified for those two STH infections and adjusted for age and sex which could affect malaria infection [47], and for location found in our analysis as confounding factor. The result shows that when considered only schistosomiasis infection, there is a trend on the risk of being infected with *P*. *falciparum*. But, in combination with trichuriasis or hookworm infection, schistosomiasis clearly increases the risk of being infected with *P*. *falciparum*. This result shows that *S*. *haematobium* alone does not predispose to *P*. *falciparum* infection in children instead of combined effect. We hypothesize that the cumulative effects of *Schistosoma*, *Trichuris* and Hookworm infections on *P*. *falciparum* parasite carriage acts at the immunological level. A potential immuno-modulation effect of a poly-parasitism not measured in our study could explain the combined effect of helminths we have observed. Indeed, there is evidence that Schistosoma infection can modulate the immune system in response to *P*. *falciparum* [9,11]. There is also evidence that *T*. *trichiura* can exert an influence on the immune response, and for instance negatively affect the antibody response to malaria vaccine candidate in children [17]. However, these potential cumulative effects of helminth infections on plasmodium infection need to be properly investigated at immunological level.

The overall incidence of malaria was 0.51 per person-year. This incidence was higher among people infected with *S*. *haematobium* compared to the uninfected, suggesting that schistosomiasis infection increases the risk of developing malaria. Children infected with *S*. *haematobium* infection had 1.4 times the risk of developing malaria than uninfected.

Regarding time-to-first malaria infection, we found that malaria occurred earlier in participants infected with Schistosoma than those uninfected even after adjustment for age, sex and location. The main symptom we have considered to define malaria was fever, which is one of the results of some endogenous pyrogen molecules activities, notably pro-inflammatory cytokine TNF-α during the infection. Some authors reported that during malaria infection, the production of pro-inflammatory cytokines as well as of anti-inflammatory cytokines can be affected by co-infection with schistosomiasis infection in an age-dependent manner [11,48]. In our study population, the assessment of the delay-to-malaria in relation to age group shows that there is no difference in terms of delay in time-to-first malaria in children aged from 11 to 16 years, while the difference was significant in children from 6 to 10 years. Children aged from 6 to 10 years infected with *S*. *haematobium* developed malaria earlier than those without *S*. *haematobium* infection. This finding could support the possible effect of age on the immune responses of malaria in co-infected subjects. However, since the finding is based on statistical significance, biological assessment is suitable for confirmation.

We can retain that exposure to schistosomiasis enhances incidence of, and susceptibility to develop malaria in our study population. This finding corroborates with previous reports like the one by Sokhna and collaborators who reported an increased in susceptibility to developing malaria in co-infected children, even though it was only in children excreting high *S*. *mansoni* eggs loads [28]; supporting therefore the hypothesis that schistosomiasis negatively affects the outcome of malaria. This stands in opposition to the idea that schistosomiasis possibly improves the outcome of malaria. Indeed, it was reported, for instance, that protection from malaria is conferred by asymptomatic *P*. *falciparum* infection or co-infection with *S*. *haematobium* in a Malian study cohort [15]. In the study presented here, we assessed the effect of having been *S*. *haematobium*-infected on malaria instead of becoming infected at time of malaria, which could affect our conclusion compared to the studies mentioned above. On the other hand, it has been shown that STH can affect susceptibility to malaria infection by acting at the immunological level [49,50]. Not having considered the STH status of participants in our analysis could have affected our results; however, since all participants were assessed and infected ones were treated for STH at inclusion, we assume that the effect of STH was minimized. The prevalence of STH was similar between the both study groups at baseline, and the STH treatment effect was considered as equally distributed between groups.

We have assessed the effect of schistosomiasis on clinical and parasitological aspects of *P*. *falciparum* infection based on prevalence of *P*. *falciparum* parasite carriage and malaria incidence. We therefore grouped our study population in relation to the schistosomiasis status. If it was easy at baseline to discriminate children infected or non-infected with *S*. *haematobium*, the problem we faced during the follow-up phase was to appropriately group our population in accordance with schistosomiasis status. Subjects infected at baseline or during the follow-up phase were treated systematically. However, they were considered as Schistosoma-infected for the whole follow-up phase and those who were not found positive throughout the survey were considered as non-infected. This approach was sustained by the fact that schistosomiasis is known as a chronic infection and, in areas where schistosomiasis is prevalent, the risk factors as playing habits, swimming, taking baths, washing clothes, distance from river are usually constant [51,52] and therefore the probability to be re-infected after treatment is high [53]. Thus, we have assessed the effect of schistosomiasis infection on *P*. *falciparum* parasite carriage at baseline and on malaria infection during the follow-up study phase.

An earlier conducted study in the same population showed that PCR has a better sensitivity than microscopy for the detection of *P*. *falciparum* parasites [9]. We have used the light microscopy Lambaréné method for the detection of *P*. *falciparum* parasites as it is the clinical gold standard. However, this may lead to potential misclassification of the participants regarding *P*. *falciparum* status at baseline. We think that if prevalence of *P*. *falciparum* carriage could be underestimated, this potential misclassification of participants would have been equally distributed in both groups and would not therefore affect the trend of our results.

This study confirms that the transmission of schistosomiasis is not evenly distributed in the vicinity of Lambaréné. Schistosomiasis infection is present in many villages but the prevalence varies significantly from one point to another. For example, we found a moderate prevalence for Bindo and Makouké villages where 19% of our study participants were found to be positive when compared to Zilé-PK villages, where 45% of our study participants were found to be positive. This corroborates with a previous pilot study conducted in the same population in 2012. The earlier-indicated prevalences of 15% and 43% for Bindo and Zilé-PK villages, respectively [14], suggest that prevalence of schistosomiasis infection is stable on each location. It was suggested that the difference observed could be explained by the fact that in Zilé-PK villages, streams represent the first source of water compared to Bindo village. The same observation could be applied to Makouké village where piped water is available for the majority of the population. Indeed, the lack of pipe water supply observed in the PK area promotes daily open freshwater contact by the population for household activities, bathing and playing, using the streams well known as schistosomiasis foci. In addition to humans, other ecological factors influence Schistosoma host snail density [54], which affect schistosomiasis prevalence. Therefore, we can assume that such factors may also sustain the difference of prevalence for schistosomiasis observed between the both locations, which requires further research. On the other hand, we have observed that areas where *S*. *haematobium* prevalence is high, a high prevalence of *P*. *falciparum* carriage was also found. Indeed, the difference in prevalence observed in favour of the PK area for *S*. *haematobium* infection was also observed for *P*. *falciparum*. This observation suggest a correlation of factors affecting both infections as either a consequence of the presence of same environmental risk factors. Another explanation could be indeed the effect of *S*. *haematobium* infection on *P*. *falciparum* infection, as demonstrated above. However, this need to be more investigated.

In summary, this study demonstrates that *S*. *haematobium* infection alone does not increase the risk of being infected with *P*. *falciparum* parasite but when associated with STH particularly with *T*. *trichiura* and hookworm, the risk does increase. On the other hand, in people exposed to schistosomiasis infection, risk and susceptibility of developing a malaria event increase in an age-dependent manner. Our results suggest that Schistosoma and probably STH co-infections in general cumulatively impact on malaria outcome in school-age children and therefore need to be accounted for when designing malaria control programs. Thus, in areas of co-endemicity and in support of higher efficiency, STH and schistosomiasis control should be considered as an additional tool of malaria control.

## Supporting information

S1 Checklist STROBE checklist. (PDF)

S1 Dataset (XLSX)
