# Supplementary material for: Schistosoma haematobium effects on Plasmodium falciparum infection modified by soil-transmitted helminths in school-age children living in rural areas of Gabon
Source: PLoS Negl Trop Dis. 2018 Aug 6;12(8):e0006663. doi: 10.1371/journal.pntd.0006663 (PMC6095623; doi:10.1371/journal.pntd.0006663)
Supplement: S1 Checklist — (PDF) [file pntd.0006663.s001.pdf]

STROBE Statement—Checklist of items that should be included in reports of ***cohort studies***

	Item No	Recommendation
**Title and abstract**	1	(*a*) Indicate the study's design with a commonly used term in the title or the abstract<br>**-We indicated the study design in the abstract**
		(*b*) Provide in the abstract an informative and balanced summary of what was done and what was found<br>**-We described the study method and main finding in the "Methodology/Principal findings section" of the abstract**
**Introduction**		
Background/rationale	2	Explain the scientific background and rationale for the investigation being reported<br>**The scientific background and the rational for the investigation are described in "Introduction section"**
Objectives	3	State specific objectives, including any prespecified hypotheses<br>**-The main objective of the study is described in "Introduction section, paragraph 2 "**
**Methods**		
Study design	4	Present key elements of study design early in the paper<br>**-The key elements of the study design are presented in "Methods section" and more details are provided in the "study procedure section, paragraph 1"**
Setting	5	Describe the setting, locations, and relevant dates, including periods of recruitment, exposure, follow-up, and data collection<br>**-We described the setting, locations and relevant dates of the study in the "Methods, Study site section"**
Participants	6	(*a*) Give the eligibility criteria, and the sources and methods of selection of participants. Describe methods of follow-up<br>**-Criteria for eligibility, method of selection and method of follow-up of participants are described in "Methods section"**
		(*b*) For matched studies, give matching criteria and number of exposed and unexposed
Variables	7	Clearly define all outcomes, exposures, predictors, potential confounders, and effect modifiers. Give diagnostic criteria, if applicable<br>**-We defined the study outcome and exposure in the "methods section"**
Data sources/ measurement	8*	For each variable of interest, give sources of data and details of methods of assessment (measurement). Describe comparability of assessment methods if there is more than one group<br>**-We described data sources and details of methods of assessment in "Methods, Sample collection and laboratory assays section"**
Bias	9	Describe any efforts to address potential sources of bias<br>**-We described methods to address potential bias in the "Methods, statistical analysis section"**
Study size	10	Explain how the study size was arrived at<br>**-We explained how the study size was arrived at in "Methods, study procedure section"**
Quantitative variables	11	Explain how quantitative variables were handled in the analyses. If applicable, describe which groupings were chosen and why<br>**-We described how the study groups were considered in "Methods section"**
Statistical methods	12	(*a*) Describe all statistical methods, including those used to control for confounding<br>**-All statistical analysis are described in "Methods, statistical analysis"**

		(*b*) Describe any methods used to examine subgroups and interactions
		(*c*) Explain how missing data were addressed
		(*d*) If applicable, explain how loss to follow-up was addressed
		(*e*) Describe any sensitivity analyses
**Results**		
Participants	13*	(a) Report numbers of individuals at each stage of study—eg numbers potentially eligible, examined for eligibility, confirmed eligible, included in the study, completing follow-up, and analysed<br>**-The number of individuals at each stage of study is reported in "Results, Study population at baseline section" and in "Fig1"**
		(b) Give reasons for non-participation at each stage<br>**Reasons for non-participation in the study for some participants are provided in "Results , Study population at baseline section" and in "Fig1"**
		(c) Consider use of a flow diagram<br>**-We have used a flow diagram "Fig1", to describe the flow of the participants in the study**
Descriptive data	14*	(a) Give characteristics of study participants (eg demographic, clinical, social) and information on exposures and potential confounders<br>**-We described the characteristics of the study population in the "Results section, Table1 and Table3"**
		(b) Indicate number of participants with missing data for each variable of interest<br>**-The number of participants with missing data is indicated for each variable of interest in the "result section"**
		(c) Summarise follow-up time (eg, average and total amount)<br>**-We provided follow-up time of the participants according to the study groups in "Result section, Table6"**
Outcome data	15*	Report numbers of outcome events or summary measures over time<br>**-We reported the numbers of outcome according to the phase of the study in "result section, Table 2 and Table 6"**
Main results	16	(*a*) Give unadjusted estimates and, if applicable, confounder-adjusted estimates and their precision (eg, 95% confidence interval). Make clear which confounders were adjusted for and why they were included<br>**-We presented unadjusted estimates, adjusted analysis, and stratified analysis and provide the reason in the "Results section. We also presented the precision of our analysis in the "Results section"**
		(*b*) Report category boundaries when continuous variables were categorized<br>**-We reported category boundaries of categorized continuous variables in "Result section, Table1"**
		(*c*) If relevant, consider translating estimates of relative risk into absolute risk for a meaningful time period
Other analyses	17	Report other analyses done—eg analyses of subgroups and interactions, and sensitivity analyses<br>**-We reported other analysis done in "Result section, Table5"**
**Discussion**		
Key results	18	Summarise key results with reference to study objectives<br>**-We summarized the key results in "Discussion section"**
Limitations	19	Discuss limitations of the study, taking into account sources of potential bias or imprecision. Discuss both direction and magnitude of any potential bias<br>**-We have discussed limitations of the study in the "Discussion section,**

		**paragraphs 4 and 5 ”**
Interpretation	20	Give a cautious overall interpretation of results considering objectives, limitations, multiplicity of analyses, results from similar studies, and other relevant evidence **-We compared our results with similar studies and discussed limitations in “Discussion section”**
Generalisability	21	Discuss the generalisability (external validity) of the study results
**Other information**		
Funding	22	Give the source of funding and the role of the funders for the present study and, if applicable, for the original study on which the present article is based **-We have provided the source of funding and the role of the founders.**

*Give information separately for exposed and unexposed groups.

Note: An Explanation and Elaboration article discusses each checklist item and gives methodological background and published examples of transparent reporting. The STROBE checklist is best used in conjunction with this article (freely available on the Web sites of PLoS Medicine at http://www.plosmedicine.org/, Annals of Internal Medicine at http://www.annals.org/, and Epidemiology at http://www.epidem.com/). Information on the STROBE Initiative is available at http://www.strobe-statement.org.
